# Randomized Trial of Oxygen Saturation Targets during and after Resuscitation and Reversal of Ductal Flow in an Ovine Model of Meconium Aspiration and Pulmonary Hypertension

**DOI:** 10.3390/children8070594

**Published:** 2021-07-14

**Authors:** Amy L. Lesneski, Payam Vali, Morgan E. Hardie, Satyan Lakshminrusimha, Deepika Sankaran

**Affiliations:** 1 Department of Stem Cell Research, University of California, Davis, Sacramento, CA 95817, USA; allesneski@ucdavis.edu; 2 Department of Pediatrics, University of California, Davis, Sacramento, CA 95817, USA; pvali@ucdavis.edu (P.V.); mehardie@ucdavis.edu (M.E.H.); dsankaran@ucdavis.edu (D.S.)

**Keywords:** meconium aspiration, oxygen saturation targets, neonatal resuscitation, persistent pulmonary hypertension of the newborn, asphyxia, ductus arteriosus, pulmonary blood flow, post-resuscitation

## Abstract

Neonatal resuscitation (NRP) guidelines suggest targeting 85–95% preductal SpO_2_ by 10 min after birth. Optimal oxygen saturation (SpO_2_) targets during resuscitation and in the post-resuscitation management of neonatal meconium aspiration syndrome (MAS) with persistent pulmonary hypertension (PPHN) remains uncertain. Our objective was to compare the time to reversal of ductal flow from fetal pattern (right-to-left), to left-to-right, and to evaluate pulmonary (Q_PA_), carotid (Q_CA_)and ductal (Q_DA_) blood flows between standard (85–94%) and high (95–99%) SpO_2_ targets during and after resuscitation. Twelve lambs asphyxiated by endotracheal meconium instillation and cord occlusion to induce MAS and PPHN were resuscitated per NRP guidelines and were randomized to either standard (85–94%) or high (95–99%) SpO_2_ targets. Out of twelve lambs with MAS and PPHN, six each were randomized to standard and high SpO_2_ targets. Median [interquartile range] time to change in direction of blood flow across the ductus arteriosus from right-to-left, to left-to-right was significantly shorter with high SpO_2_ target (7.4 (4.4–10.8) min) compared to standard SpO_2_ target (31.5 (21–66.2) min, *p* = 0.03). Q_PA_ was significantly higher during the first 10 min after birth with higher SpO_2_ target. At 60 min after birth, the Q_PA_, Q_CA_ and Q_DA_ were not different between the groups. To conclude, targeting SpO_2_ of 95–99% during and after resuscitation may hasten reversal of ductal flow in lambs with MAS and PPHN and transiently increase Q_PA_ but no differences were observed at 60 min. Clinical studies comparing low and high SpO_2_ targets assessing hemodynamics and neurodevelopmental outcomes are warranted.

## 1. Introduction

Successful transition of the fetus to extrauterine life involves a rapid increase in pulmonary blood flow (Q_PA_) during the first few breaths after birth, allowing the lungs to establish as the site of gas exchange. A failure of this transition can lead to persistent pulmonary hypertension of the newborn (PPHN), characterized by sustained elevation of pulmonary vascular resistance (PVR), right-to-left shunting of blood across the foramen ovale and ductus arteriosus, and reduced Q_PA_ [1]. These newborns experience severe respiratory distress and labile hypoxemia soon after birth.

Oxygen (O_2_) mediates decrease in PVR after birth and has been used to correct the hypoxemia in PPHN, along with strategies to improve lung inflation with respiratory support [2,3]. Current neonatal resuscitation guidelines recommend initiating ventilation with 21% O_2_ with subsequent O_2_ titration to target goal preductal pulse oximetry O_2_ saturation (SpO_2_) ranges corresponding to the minute of life, to achieve 85–95% SpO_2_ by 10 min after birth. This strategy has been associated with optimal hemodynamics and gas exchange during resuscitation [4]. Rawat et al. reported that targeting 95–99% SpO_2_ in the post-resuscitation period lowered PVR and improved cerebral O_2_ delivery, while targeting 85–89% SpO_2_ increased PVR, and decreased Q_PA_ and cerebral O_2_ delivery in a term ovine model of meconium aspiration syndrome (MAS) with PPHN [5]. However, flow across the ductus arteriosus (Q_DA_) was not evaluated in this study and ductal flow is a major contributor to Q_PA_ [6]. The optimal preductal SpO_2_ target range during resuscitation and post-resuscitation period that hastens reversal of shunting across the patent ductus arteriosus (PDA) from the fetal pattern to the postnatal pattern of left-to-right remains unknown.

We hypothesized that the time to reversal of shunting across the PDA is shorter with high SpO_2_ target of 95–99% compared to standard SpO_2_ target of 85–94%. Our objective was to compare the time to reversal of shunt across the PDA from the fetal (right-to-left) to the postnatal (left-to-right) pattern between standard (85–94%) and high (95–99%) SpO_2_ target ranges during resuscitation and the post-resuscitation period in a term ovine asphyxiated model of MAS and PPHN. We also evaluated the changes in Q_PA_, Q_DA_ and carotid blood flow (Q_CA_), and gas exchange at 10 min and 60 min after birth between low and high SpO_2_ targets as secondary outcomes.

## 2. Materials and Methods

The protocol was approved by the Institutional Animal Care and Use Committee (IACUC, protocol #20267) at the University of California Davis, CA, USA. This protocol involves a perinatal model of MAS and PPHN in term newborn lambs that has been extensively described previously [5,7]. All experiments were performed in accordance with animal ethical guidelines (ARRIVE) [8]. Time-dated near-term (139–141 days) gestation pregnant ewes from Van Laningham Farm (Arbuckle, CA, USA) underwent cesarean section following overnight fasting, after endotracheal intubation under general anesthesia with IV diazepam and ketamine, and inhaled 2% isoflurane, as previously described [9].

### 2.1. Fetal Instrumentation

The fetal lamb was partially exteriorized and intubated with a 4.5-mm cuffed endotracheal tube (ETT), the lung fluid was passively drained by gravity, and the ETT was occluded to prevent entry of air. The lamb was instrumented under maternal anesthesia after subcutaneous bupivacaine infiltration. Catheters were inserted into the right carotid artery and right jugular vein for preductal arterial blood draws, invasive blood pressure and heart rate monitoring, and IV access respectively. A flow probe (Transonics, Ithaca, NY, USA) was placed around the left carotid artery to measure blood flow. A left thoracotomy was performed, and flow probes were placed around the left pulmonary artery and ductus arteriosus to measure blood flows. Subsequently, the thoracotomy and neck incisions were closed in layers. The baseline hemodynamics were recorded and arterial blood gases were obtained.

### 2.2. Meconium Instillation, Asphyxia and Resuscitation

Fetal lambs were asphyxiated following instrumentation by umbilical cord occlusion (by manual compression) for 5 min or until heart rate decreased below 40 beats per minute. Meconium (5 mL/kg of 20% meconium suspended in ewe amniotic fluid) was simultaneously instilled into their endotracheal tube as previously described [7,10]. During asphyxiation, the lambs gasped and spontaneously aspirated the meconium into their lungs. The cord compression was released for 2 min to allow hemodynamic recovery, followed by another 5-min cord occlusion.

Lambs were randomized to standard SpO_2_ target (85–94%) or high SpO_2_ target (95–99%) using opaque envelopes prior to the beginning of the study (incision for cesarean section). The lambs were then delivered and ventilated with peak inflation pressures (PIP) of 30–35 cm H_2_O, PEEP of 5 cm H_2_O, rate of 40 breaths per minute and inspired O_2_ of 21% that was then titrated based on preductal SpO_2_ per Neonatal Resuscitation Program (NRP) guidelines during resuscitation to achieve a goal of 85–94% (standard target arm) [11] or titrated to achieve a goal of 95–99% SpO_2_ after delivery (high target arm). The titration of oxygen was proportional to the difference between observed SpO_2_ and target SpO_2_ and was performed every minute. The endotracheal tube was connected to a ventilator and PaCO_2_ was targeted in the 40–60 mm Hg range to allow permissive hypercapnia. The resuscitators were not blinded to the intervention. Hemodynamics were continuously monitored, and arterial blood gases were obtained at baseline, 10-min and subsequently at 15–min intervals. Lambs were monitored for up to 60 min and were finally euthanized using IV pentobarbital (Fatal-Plus, Vortech Pharmaceuticals, Dearborn, MI, USA).

### 2.3. Primary and Secondary Outcomes

Primary outcome measures were time to reversal of ductal shunt (from right-to-left to left-to-right) from the time of delivery.

Secondary outcome measures were changes in Q_PA_, Q_DA_ and Q_CA_, and gas exchange at 10 min and 60 min after birth between standard and high SpO_2_ targets. Cerebral oxygen delivery (mL/kg/min) was calculated by multiplying carotid artery oxygen content (CaO_2_ = (1.34 × Hemoglobin in g/dL × SaO_2_%/100%) + (partial pressure of O_2_ in mm Hg × 0.0031)) and left carotid artery blood flow (mL/kg/min).

### 2.4. Data Collection and Analysis

Hemodynamic variables were continuously monitored and recorded using BIOPAC systems data acquisition software (Goleta, CA, USA). Blood gases were analyzed using a blood gas analyzer (Radiometer ABL90 FLEX, Denmark). By convention, the right-to-left direction of Q_DA_ was labeled as negative and left-to-right direction of Q_DA_ was labeled as positive. Categorical data were analyzed using chi-squared test with Fisher’s exact test as appropriate, parametric continuous data were analyzed using unpaired *t*-test, and changes in Q_DA_ and Q_PA_ over time were compared using repeated measures ANOVA. The median time to reversal of shunting (non-parametric) was compared between standard and high SpO_2_ targets using Wilcoxon rank sum test. Statistical significance was defined as *p* < 0.05.

## 3. Results

Out of twelve near-term lambs that were asphyxiated, six were randomized to standard SpO_2_ target and the remaining six were randomized to high SpO_2_ target. Hemodynamic and arterial blood gas characteristics at fetal baseline prior to asphyxia were similar between the study groups (Table 1).

### 3.1. Time to Reversal of Shunt across the PDA

**Figure 1:**
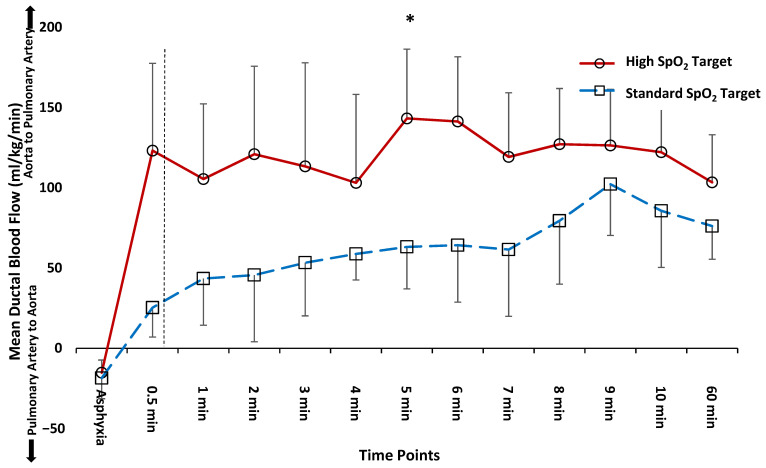
Mean ductus arteriosus blood flow (Q_DA_) and direction of flow over timepoints from asphyxia, 0.5 min and up to 60 min after delivery between the two oxygen target groups. Delivery of lamb is indicated by the dashed, vertical line. Positive flow indicates left-to-right shunting from aorta to the pulmonary artery. Negative flow indicates right-to-left shunting from pulmonary artery to the aorta. Median (interquartile range) time for exclusive left-to-right ductal shunting post-delivery was significantly shorter in the high SpO_2_ target group (median 7.4 vs. 31.5 min, by Wilcoxon rank sum test). * *p* < 0.05, left-to-right ductal flow was significantly higher with high SpO_2_ target compared to standard SpO_2_ target (*p* = 0.002, ANOVA repeated measures). However, by 60 min after delivery, the Q_DA_ was not different between the two SpO_2_ targets. The QDA significantly increased in both high and standard SpO_2_ targets from fetal baseline to 5-min after birth, and significantly decreased by 60 min after birth in the high SpO_2_ target.

Median (interquartile range) time to transition from right-to-left to exclusive left-to-right shunting across the ductus arteriosus was significantly shorter with high SpO_2_ target (7.4 (4.4–10.8) min) compared to standard SpO_2_ target (31.5 (12–66.2) min, *p* = 0.03, Figure 1). The mean Q_DA_ (left-to-right) flow was increased with the high SpO_2_ targets from 0.5 to 10 min by ANOVA repeated measures. The Q_DA_ significantly increased from fetal baseline to 5-min after birth in both standard and high SpO_2_ targets (*p* < 0.05) and decreased significantly by 60 min in high SpO_2_ target (*p* < 0.05).

### 3.2. Comparison of Hemodynamics and Arterial Blood Gas Parameters at 5 and 10 min after Birth

**Figure 2:**
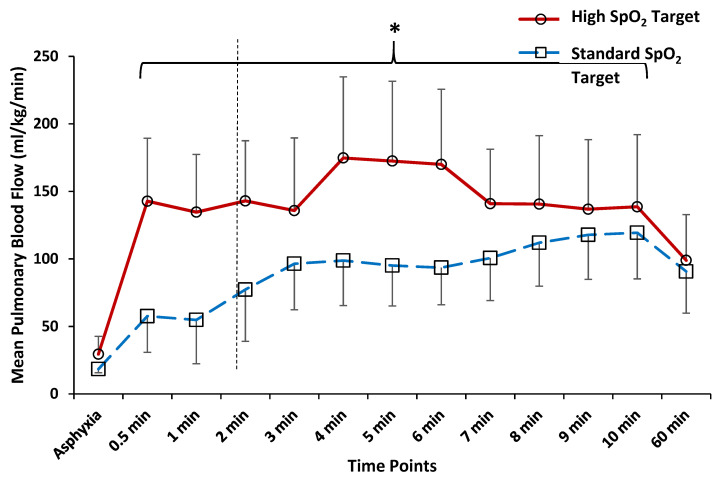
Mean pulmonary blood flow (Q_PA_) during the immediate post-natal period in a lamb model of meconium aspiration syndrome (MAS) and persistent pulmonary hypertension (PPHN). Delivery of lamb is indicated by the dashed, vertical line. The Q_PA_ was significantly higher from 0.5 min to 10 min after delivery with high SpO_2_ target compared to standard SpO_2_ target (*p* = 0.048). However, by 60 min after delivery, the Q_PA_ was not different between the two SpO_2_ targets. * *p* < 0.05.

During the first 10 min after delivery, the Q_DA_ (left-to-right, Figure 1, *p* = 0.002) and Q_PA_ (Figure 2, *p* = 0.048) were significantly higher with high SpO_2_ target compared to standard SpO_2_ target by ANOVA repeated measures. The Q_PA_ significantly decreased from 5-min to 60-min after birth with high SpO_2_ target. The hemodynamic parameters at 5 min and at 10 min time points after delivery are depicted in Table 2 and Table 3 respectively and were not different. The arterial blood gas parameters were not different at 10-min after delivery (Table 3). Although the PaO_2_ and SpO_2_ were higher in the high SpO_2_ target group, these differences did not reach statistical significance.

### 3.3. Comparison of Hemodynamics and Arterial Blood Gas Parameters at 60 min after Birth

There was no significant difference in mean Q_CA_, Q_DA_, or Q_PA_ blood flows at 60 min after birth (Table 4). One lamb randomized in the standard SpO_2_ group maintained a bidirectional Q_DA_ shunt throughout the study period. There were no significant differences in inspired O_2_ concentration, arterial O_2_ content, cerebral O_2_ delivery or PaO_2_ between the standard and high SpO_2_ target groups at 60 min after birth. However, the pH was higher (*p* = 0.046) and PaCO_2_ was lower (*p* = 0.042) with high SpO_2_ target at the 60 min timepoint.

**Figure 3:**
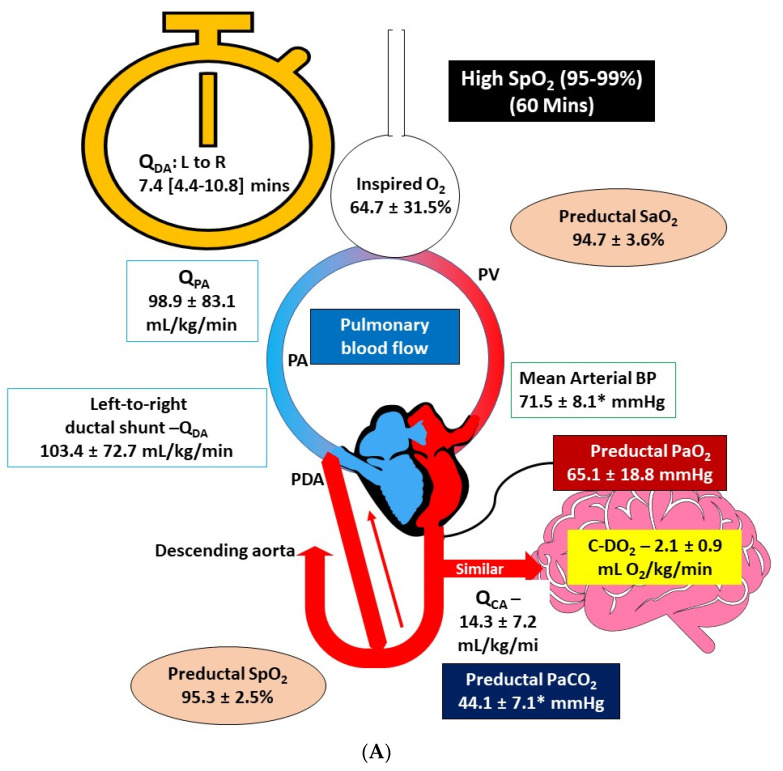

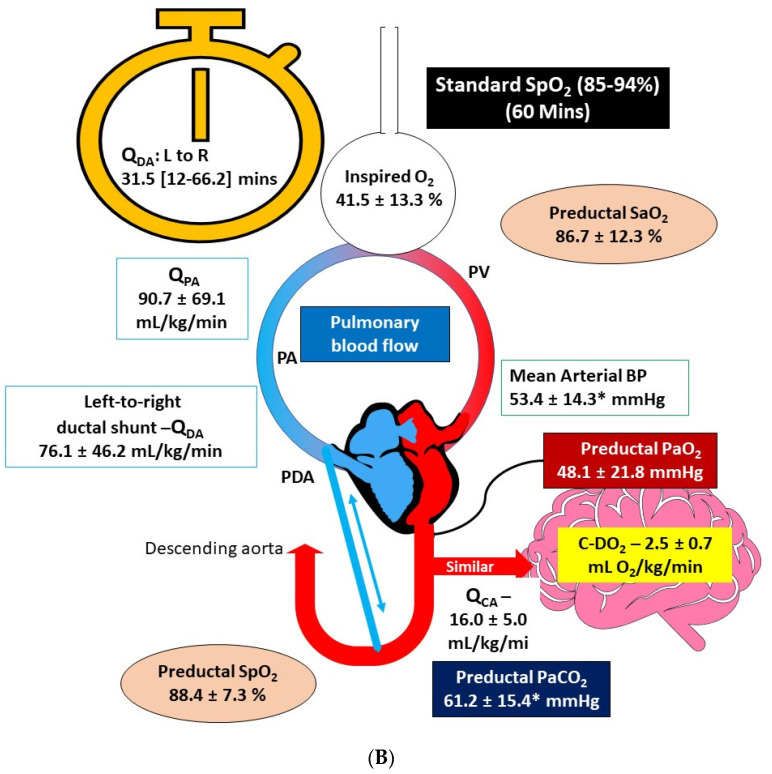
(**A**) Graphical summary of the results demonstrating the hemodynamics and gas exchange at 60 min after birth with high (95–99%) oxygen saturation (SpO_2_) targets in an ovine model of meconium aspiration and pulmonary hypertension. Median time to reversal of shunting was 7.4 (4.4–10.8) min after birth. The inspired oxygen concentration, preductal SpO_2_ and arterial oxygen saturation (SaO_2_), left-to-right ductal shunting (Q_DA_), left pulmonary artery blood flow (Q_PA_), left carotid artery blood flow (Q_CA_), cerebral O_2_ delivery (C-DO_2_) and arterial partial pressure of carbon dioxide (PaCO_2_) are shown. Shorter time to reversal of Q_DA_ shunt to left-to-right, transiently increased Q_DA_ and Q_PA_ from 0.5–10 min after birth, and lower PaCO_2_ at 60 min were observed with high SpO_2_ target. * *p* < 0.05 compared to standard SpO_2_ target group in (**B**). (**B**) Graphical summary of the results demonstrating the hemodynamics and gas exchange at 60 min after birth with standard (85–94%) oxygen saturation (SpO_2_) targets in an ovine model of meconium aspiration and pulmonary hypertension. Median time to reversal of shunting was 31.5 (12–66.2) min after birth. The inspired oxygen concentration, preductal SpO_2_ and arterial oxygen saturation (SaO_2_), left-to-right ductal shunting (Q_DA_), left pulmonary artery blood flow (Q_PA_), left carotid artery blood flow (Q_CA_), cerebral O2 delivery (C-DO_2_) and arterial partial pressure of carbon dioxide (PaCO_2_) are shown. Longer time to reversal of Q_DA_ shunt to left-to-right and higher PaCO_2_ at 60 min were observed with standard SpO_2_ target. * *p* < 0.05 compared to high SpO_2_ target group shown in (**A**).

An illustration summarizing the hemodynamics and gas exchange at 60 min after birth is presented as Figure 3A,B.

## 4. Discussion

Neonatal PPHN is commonly associated with parenchymal lung disease such as MAS [1]. Mortality rates of 4–33% have been reported for neonatal PPHN [12]. Right-to-left shunting via the PDA and PFO resulting in labile hypoxemia is characteristic of PPHN. Supplemental O_2_ can decrease PVR thus improving Q_PA_, hastening reversal of extrapulmonary shunting to left-to-right. We demonstrate quicker reversal of ductal shunting to left-to-right with higher (95–99%) compared to the lower (85–94%) SpO_2_ target during resuscitation and in the post-resuscitation period.

The American Academy of Pediatrics (AAP) NRP Textbook of Neonatal Resuscitation recommends initiating resuscitation with 21% O_2_, titrating inspired O_2_ to achieve the target SpO_2_ range for every minute, and finally 85–95% by 10 min after birth. Maintaining SpO_2_ within the range recommended by NRP by actively titrating the inspired O_2_ led to effective oxygenation and Q_PA_ in an ovine model of MAS [4]. However, there is wide variation among neonatologists in their preference of SpO_2_ targets immediately after the initial resuscitation period [13,14]. In a recent animal study by Rawat et al., higher SpO_2_ target of 95–99% resulted in lower PVR, higher Q_PA_, higher cerebral O_2_ delivery with lower lactate levels, but was associated with higher inspired O_2_ and higher lung 3-nitrotyrosine, a marker of oxidative stress compared to lower ranges of target SpO_2_ [5]. However, the authors did not evaluate direction of ductal flow and Q_DA_ in that study.

During neonatal resuscitation, O_2_ needs to be titrated judiciously to avoid hypo or hyperoxia. Kapadia et al. reported that post-resuscitation hyperoxia with perinatal acidemia is associated with higher incidence of moderate to severe hypoxic ischemic encephalopathy [15]. Thus, it is prudent to decrease supplemental O_2_ in a timely manner to avoid hyperoxemia. We show that the benefits of targeting higher SpO_2_ are transient and Q_PA_ is identical between the two study groups by 60 min after birth. We also observed lower PaCO_2_ in the high SpO_2_ target group at 60 min after birth. We speculate that the higher SpO_2_ target may have caused a transient surge in Q_PA_ (Figure 2) that might have contributed to better gas exchange and lower PaCO_2_ (Figure 3A,B). Additionally, the initial increase in left-to-right Q_DA_ and increase in Q_PA_ were not persistent at 60 min after birth (Figure 1 and Figure 2). We speculate that reduction in Q_PA_ could be secondary to transient effect of increased inspired O_2_ on PVR or ductal constriction limiting Q_DA_ leading to lower Q_PA_. As the ductal flow remained steady between 5 min and 60 min after birth (Figure 1), we suspect that the PDA remained patent at 60 min in these lambs. We speculate that higher SpO_2_ target is associated with earlier ductal narrowing and less cardiac dysfunction with quicker cardiovascular recovery from the asphyxial insult resulting in better mean arterial pressures (MAPs). Whereas in the standard SpO_2_ target group, there may be slower ductal constriction and cardiovascular recovery from the asphyxial insult resulting in lower MAPs. Higher volume of shunt from the aorta-to-pulmonary artery is likely to expose ductal tissue to higher PaO_2_ and hasten narrowing of the ductus. Furthermore, the diastolic BP were higher with high SpO_2_ target at 60 min after delivery when compared to standard SpO_2_ target (65 ± 13 vs. 45 ± 6, *p* = 0.02), possibly due to ductal constriction, thus contributing to higher MAPs.

Our study has several limitations. The severity of asphyxia was mild to moderate. Despite this, we did demonstrate significant degree of hypoxemia, low Q_PA_ and right-to-left shunting across the PDA following delivery in all the lambs (Table 1 and Table 2 and Figure 1). We did not measure the pulmonary artery and left atrial pressures, and hence could not evaluate the PVR. In addition, we did not evaluate the shunting across the PFO. We had a small sample size and larger number of lambs might have led to different results. The markers of oxidative stress have not been evaluated. We did not recover the lambs and assess for long term outcomes. Although we targeted to achieve preductal SpO_2_ within a narrow set range, the achieved SpO_2_ was 88 ± 7% and 95 ± 3% in the standard and high SpO_2_ target groups, respectively. This difficulty in achieving a set SpO_2_ target range has previously been demonstrated in randomized trials in preterm infants [16,17].

To our knowledge, this is the first comparison of time to reversal of ductal shunting in PPHN between standard and high SpO_2_ targets during resuscitation and in the post-resuscitation period. The novel aspect of this study is the measurement of Q_DA_ along with the direction of flow in a large mammalian model of MAS and PPHN closely mimicking human cardiopulmonary physiology. The lambs underwent meconium aspiration by spontaneous aspiration with negative pressure in the perinatal period during transition rather forceful instillation of meconium in the postnatal (1–3 days, often the case in piglet models) period [18]. Although an ovine model of PPHN can be induced by prenatal ductal ligation, we are unable to assess Q_DA_ in that model. Finally, the randomized study design is a major strength of this study. 

## 5. Conclusions

In this term lamb model of MAS and PPHN, targeting SpO_2_ at a higher range (95–99%) during resuscitation and in the immediate post-resuscitation period, led to a quicker transition to left-to-right shunting across the PDA but did not result in sustained increase in pulmonary blood flow. These findings support the current NRP recommendations to target preductal SpO_2_ between 85–95% by 10 min. Clinical trials evaluating hemodynamics and long-term neurocognitive outcomes in term neonates comparing current recommended range to higher SpO_2_ targets are warranted in patients at risk of PPHN such as MAS with asphyxia and congenital diaphragmatic hernia.

## Figures and Tables

**Table 1:** Comparison of fetal baseline hemodynamic and arterial blood gas parameters and end-asphyxia hemodynamic parameters in a near-term ovine model of meconium aspiration syndrome (MAS) and persistent pulmonary hypertension (PPHN) randomized to standard (85–94%) and high (95–99%) preductal SpO_2_ target groups.

Parameter	Standard SpO_2_ Target (85–94%, n = 6)	High SpO_2_ Target (95–99%, n = 6)
Weight (kg)	3.4 ± 0.8	3.3 ± 0.4
Gestational Age (days)	139.7 ± 0.7	139.3 ± 0.8
Parameters at Fetal Baseline		
Hemoglobin, g/dL	13.88 ± 2.47	11.93 ± 1.52
pH	7.18 ± 0.08	7.19 ± 0.06
PaCO_2_ (mm Hg)	74.73 ± 12.58	64.95 ± 5.17
PaO_2_ (mm Hg)	22.08 ± 6.22	28.38 ± 4.51
Cerebral Oxygen Delivery (mL/kg/min)	4.26 ± 2.17	2.40 ± 0.64
Lactate (mmol/L)	2.20 ± 0.53	2.37 ± 0.96
Heart Rate (bpm)	155.17 ± 21.95	164.23 ± 21.50
Mean Arterial Blood Pressure (mm Hg)	55.52 ± 4.42	61.11 ± 3.8
Mean Ductal Blood Flow (mL/kg/min)	−125.89 ± 64.77	−84.33 ± 36.09
Mean Pulmonary Artery Blood Flow (mL/kg/min)	25.21 ± 8.25	49.39 ± 23.59
Duration of Asphyxia (min)	13.43 ± 0.63	15.32 ± 1.82
Parameters at End of Asphyxia		
Mean Carotid Artery Blood Flow (mL/kg/min)	24.0 (5.5)	21.2 (4.4)
Mean Ductal Blood Flow (mL/kg/min)	−18.3 (6.9)	−15 (7.7)
Mean Pulmonary Artery Blood Flow (mL/kg/min)	18.3 (2.7)	29.4 (13.2)

Data presented as mean ± standard deviation. Data were not different by unpaired *t* test. PaCO_2_ = arterial carbon dioxide pressure; PaO_2_ = arterial oxygen pressure; SpO_2_ = oxygen saturation.

**Table 2:** Comparison of hemodynamics at 5 min after birth in a perinatal lamb model of MAS and PPHN.

Parameter	Standard SpO_2_ Target (85–94%, n = 6)	High SpO_2_ Target (95–99%, n = 6)
Heart Rate (bpm)	182.12 ± 12.14	170.78 ± 13.08
Mean Arterial Blood Pressure (mm Hg)	52.91 ± 18.79	65.38 ± 8.81
Mean Carotid Flow (Q_CA_, mL/kg/min)	34.10 ± 14.84	20.22 ± 8.47

Data presented as mean and standard deviation. Positive flow indicates left-to-right ductus arteriosus blood flow. There was no significant difference between the two SpO_2_ targets at 5-min timepoint by unpaired *t*-test.

**Table 3:** Comparison of hemodynamics and oxygenation at 10 min after birth in a perinatal lamb model of MAS and PPHN.

Parameter	Standard SpO_2_ Target (85–94%, n = 6)	High SpO_2_ Target (95–99%, n = 6)
Hemoglobin, g/dL	13.18 ± 1.95	11.9 ± 1.40
pH	7.05 ± 0.24	7.11 ± 0.19
PaCO_2_ (mm Hg)	79.48 ± 48.89	64.5 ± 42.89
PaO_2_ (mm Hg)	53.42 ± 22.97	60.13 ± 2.55
SaO_2_ (%)	89.7 ± 4.32	93.9 ± 3.96
SpO_2_ (%)	77.75 ± 30.61	91.33 ± 6.51
CaO_2_ (mL O_2_/dL)	15.98 ± 2.14	15.14 ± 1.62
Cerebral Oxygen Delivery (mL/kg/min)	4.78 ± 2.23	3.69 ± 0.47
Lactate (mmol/L)	6.02 ± 1.97	5.90 ± 1.75
Heart Rate (bpm)	176.0 ± 13.22	162.55 ± 31.18
Mean Arterial Blood Pressure (mmHg)	59.34 ± 9.497	65.67 ± 8.98
Mean Carotid Flow (Q_CA_, mL/kg/min)	29.17 ± 12.31	20.44 ± 7.32
Inspired O_2_ (%) at 10 min	40.75 ± 39.5	30.67 ± 16.74

Data presented as mean and standard deviation. No significant differences in parameters between the standard and high SpO_2_ target groups at 10-min time point by unpaired *t*-test.

**Table 4:** Comparison of hemodynamics and oxygenation comparisons at 60 min after birth in a perinatal lamb model of MAS and PPHN.

Parameter	Standard SpO_2_ Target (85–94%, n = 6)	High SpO_2_ Target (95–99%, n = 6)
Hemoglobin, g/dL	12.32 ± 3.17	11.85 ± 1.08
pH	7.12 ± 0.11	7.25 ± 0.09 ^ǂ^
PaCO_2_ (mmHg)	61.23 ± 15.42	44.08 ± 7.07 ^ǂ^
PaO_2_ (mmHg)	48.14 ± 21.76	65.08 ± 18.77
SaO_2_ (%)	86.66 ± 12.29	94.68 ± 3.55
SpO_2_ (%)	88.4 ± 7.3	95.33 ± 2.50
CaO_2_ (mlO_2_/dL)	14.96 ± 4.22	15.23 ± 1.44
Cerebral Oxygen Delivery (ml/kg/min)	2.54 ± 0.68	2.12 ± 0.9
Lactate (mmol/L)	4.25 ± 1.94	4.07 ± 1.75
Heart Rate (bpm)	167.76 ± 32.46	154.56 ± 9.94
Mean Arterial Blood Pressure (mmHg)	53.41 ± 14.30	71.51 ± 8.14 ^ǂ^
Mean Carotid Flow (Q_CA_-mL/kg/min)	16.01 ± 4.96	14.28 ± 7.21
Inspired O_2_ (%) at 60 min	41.5 ± 13.25	64.66 ± 31.51

PaCO_2_ = arterial carbon dioxide pressure; PaO_2_ = arterial oxygen pressure; SaO_2_ = arterial oxygen saturation from blood gas; SpO_2_ = preductal pulse oximeter oxygen saturation; CaO_2_ = arterial oxygen content. ^ǂ^ Significantly different from the 85–94% target group; *p* < 0.05, unpaired student *t*-test, unequal variances.

## Data Availability

The data presented in this study are available in this article.
